# Identification of an immunodominant region on a group A *Streptococcus* T-antigen reveals temperature-dependent motion in pili

**DOI:** 10.1080/21505594.2023.2180228

**Published:** 2023-02-21

**Authors:** Jeremy M. Raynes, Paul G. Young, Natalie Lorenz, Jacelyn M.S. Loh, Reuben McGregor, Edward N. Baker, Thomas Proft, Nicole J. Moreland

**Affiliations:** a School of Medical Sciences, The University of Auckland, Auckland, New Zealand; b Maurice Wilkins Centre for Molecular Biodiscovery, The University of Auckland, Auckland, New Zealand; c School of Biological Sciences, The University of Auckland, Auckland, New Zealand

**Keywords:** *Streptococcus pyogenes*, T-antigen, pilus, antibody, temperature, motion

## Abstract

Group A *Streptococcus* (GAS) is a globally important pathogen causing a broad range of human diseases. GAS pili are elongated proteins with a backbone comprised repeating T-antigen subunits, which extend from the cell surface and have important roles in adhesion and establishing infection. No GAS vaccines are currently available, but T-antigen-based candidates are in pre-clinical development. This study investigated antibody-T-antigen interactions to gain molecular insight into functional antibody responses to GAS pili. Large, chimeric mouse/human Fab-phage libraries generated from mice vaccinated with the complete T18.1 pilus were screened against recombinant T18.1, a representative two-domain T-antigen. Of the two Fab identified for further characterization, one (designated E3) was cross-reactive and also recognized T3.2 and T13, while the other (H3) was type-specific reacting with only T18.1/T18.2 within a T-antigen panel representative of the major GAS T-types. The epitopes for the two Fab, determined by x-ray crystallography and peptide tiling, overlapped and mapped to the N-terminal region of the T18.1 N-domain. This region is predicted to be buried in the polymerized pilus by the C-domain of the next T-antigen subunit. However, flow cytometry and opsonophagocytic assays showed that these epitopes were accessible in the polymerized pilus at 37°C, though not at lower temperature. This suggests that there is motion within the pilus at physiological temperature, with structural analysis of a covalently linked T18.1 dimer indicating “knee-joint” like bending occurs between T-antigen subunits to expose this immunodominant region. This temperature dependent, mechanistic flexing provides new insight into how antibodies interact with T-antigens during infection.

## Introduction

*Streptococcus pyogenes*, or Group A *Streptococcus* (GAS), is an exclusively human, Gram-positive bacterium that causes a wide range of diseases, including pharyngitis, skin and soft tissue infection, necrotizing fasciitis, and toxic shock syndrome [1]. Post-streptococcal autoimmune sequelae include acute rheumatic fever (ARF), rheumatic heart disease (RHD), and acute glomerulonephritis (AGN) [2]. ARF and RHD rates have been decreasing in most high-income settings, but continue to cause significant morbidity and mortality in low-income regions of the world [3]. Disproportionally high rates are also reported in Indigenous communities within countries such as New Zealand and Australia [4, 5].

GAS uses a variety of adhesion factors for colonization such as lipoteichoic acid, M protein, several fibronectin-binding proteins, and pili [6, 7]. Pili (singular pilus), are thin, hair-like protrusions extending from the bacterial cell surface, which have important roles in biofilm formation and immune evasion, as well as host cell adhesion [8–11]. The GAS pilus typically comprises three structural proteins: the backbone pilin, also known as the T-antigen, and two ancillary proteins, AP1 and AP2 [12–14]. AP1 is the tip adhesin, often referred to as collagen-binding protein A (Cpa), whereas AP2 is the anchor protein that links the pilus to the cell wall [15, 16]. To facilitate formation of the multimeric pilus structure, the T-antigen contains an LPXTG motif in the C-terminal domain. This is recognized by a specific pilus-assembly sortase (SrtC) that forms amide bonds between the threonine and the ᵋ-amino group of a lysine residue on another pilin subunit enabling T-antigens to be covalently linked in an elongated fashion [13, 14]. The T-antigen was described as a **T**rypsin-resistant serological marker by Rebecca Lancefield decades prior to the discovery of GAS pili, and *tee* gene sequencing continues to be employed as a supplementary typing tool alongside standard *emm* typing (sequencing of the hypervariable region of the *emm* gene that encodes M protein) [17, 18].

The genes encoding the pilus proteins and the assembly sortases are located on an operon within the highly variable Fibronectin-T-antigen-Collagen binding protein (FCT) region. Each GAS strain carries one of the nine distinct FCT regions which encode for six antigenically different pilus types [13, 19]. Crystal structures of five backbone pilins/T-antigens (T1, T6, T18.1, T3.2, and T13) have revealed a rigid two- or three-domain immunoglobulin (Ig)-like structure with antiparallel β-strands [18, 20, 21]. The T-antigen structures are further stabilized by intramolecular isopeptide bonds within each Ig domain that form spontaneously in an autocatalytic process providing tensile strength to the pili [22].

There is currently no licenced vaccine for GAS, though several candidates are in development including those based on the M protein and “combination” vaccines that comprise conserved protein and carbohydrate antigens [23]. The protective potential of GAS pili components as vaccines was recently demonstrated by immunization of rabbits with the food-grade bacterium *Lactococcus lactis* expressing the complete M18/T18 or M28/T28 pili. This resulted in neutralizing antibodies that inhibited bacterial adhesion to epithelial cells and facilitated immune-mediated killing of bacteria via opsonophagocytosis [24]. While there is antigenic variation of the pilus proteins, this is less substantial than for the M protein, and it has been estimated that a vaccine consisting of 18 T-antigens would provide >95% coverage of global strains [25, 26]. In keeping, a multivalent T-antigen-based vaccine candidate (TeeVax) comprised three fusion proteins, each consisting of five to seven single T-antigen domains, induces antibodies that react with all major pilus types and provides protective efficacy in a murine challenge model of invasive GAS [27].

A recent structural comparison of two-domain T-antigens revealed that sequence variation is distributed along the full length of the protein and shields a highly conserved core. However, significant cross-reactive antibody responses were observed between T18.1, T18.2, T3.2, and T13 suggesting the existence of shared epitopes between selected T-antigens that may aid vaccine design [18]. The aim of this study was to explore the antibody response to T-antigens at the molecular level using T18.1 as a model T-antigen. Monoclonal antibody fragments were isolated using phage display technology [28] and, using a combination of structural and immunological analyses, an immunodominant epitope has been mapped that suggests “knee-like” flexibility between T-antigen subunits in GAS pili is able to expose this cross-reactive region.

## Methods

### Mouse and rabbit immunizations

Animal experiments were performed in the Vernon Jansen Unit at the University of Auckland using protocols approved by the University of Auckland Animal Ethics Committee (ref.1664). Female FVB/n mice (n = 5) were immunized intranasally under isoflurane anaesthesia with 10^8^ CFU live *L. lactis* PilM18 in 25 μl on days 0, 14, and 28 as described [18, 24]. Euthanasia was performed on day 52, 10 days after challenge with an M18/T18 strain (ATCC strain MGAS8232), serum was collected and spleens were harvested and snap frozen in liquid nitrogen and stored at −80°C until use. Previously generated sera from New Zealand white rabbits immunized with recombinant T18.1 was used as a control in immunoassays [18].

### Antigen preparation

The panel of 14 recombinant T-antigens used to characterize antibody reactivity has been previously described [18, 29]. All T-antigens were expressed in *Escherichia coli* BL21 (DE3) cells, and N-terminal His_6_-tags were removed using recombinant tobacco etch virus (rTEV) prior to use in immunoassays.

The recombinant T18.1 dimer was constructed by inserting the coding sequence for *tee18.1* into pProExHTA in tandem such that the vector encoded expression of two T18.1 antigens in which the C-terminal sortase motif of the first monomer is linked to the N-terminus of the following monomer, via a flexible (glycine)_3_ linker. The first five N-terminal residues from the second T18.1 monomer (ETAGV) were removed to allow the positioning of the sortase motif of the first monomer near the sortase motif peptide “binding” cleft in the second monomer. Expression and purification of the T18.1 dimer followed published protocols for T-antigen monomers [18]. Briefly, *E. coli* BL21 (DE3) cells transformed with the expression plasmid were induced with 0.3 mM isopropyl-β-D-1-thiogalactopyranoside (IPTG) and grown at 18°C for 16 h. Cell pellets were resuspended in lysis buffer (50 mM Tris-Cl [pH 8.0], 300 mM NaCl, 10 mM imidazole), lysed using a cell disruptor (Constant Cell Disruption Systems) and the protein enriched from the soluble phase using Ni^2+^-NTA affinity purification. The His_6_ affinity tag was cleaved with recombinant tobacco etch virus (rTEV) protease and rTEV-His_6_ protease, and uncleaved protein was removed by subtractive immobilized-metal affinity chromatography (IMAC). The unbound protein fraction containing recombinant T-antigen was further purified by size exclusion chromatography on a Superdex S200 10/300 column (GE Healthcare) in crystallization buffer (10 mM Tris-Cl [pH 8.0], 100 mM NaCl).

For generation of biotinylated recombinant T18.1, the *tee18.1* sequence [18] was re-cloned into a pProExHTA vector modified to encode for an N-terminal His-tag and a C-terminal Avi-tag. *E. coli* BL21 (DE3) were co-transformed with pProExHTA-*tee18.1-Avitag* and pACYC184BirA, and expression media were supplemented with 20 uM D-Biotin for in cell biotinylation. The biotinylated T18.1 was purified using Ni^2+^-NTA affinity chromatography, the His_6_-tag removed using rTEV, and final purification was performed using size exclusion chromatography as described above [18]. Successful biotin labelling was confirmed by immunoaffinity pulldown with streptavidin-coupled M-280 Dynabeads (Thermofisher) following by SDS-PAGE.

The T18.1 peptide library was designed to cover the entirety of the mature T18.1 monomer. The N-terminal signal sequence was excluded, and the library ended at the conserved threonine residue of the Sortase C recognition site (QVPTG). The tiled peptide library comprised 2915-mer peptides overlapping by five residues (Supplementary Table S1). The peptides were synthesized (GenScript) and purified to >85%.

### Fab-page display and Fc fusion generation

The amplification of antibody genes and murine Fab-phagemid library construction was based on published protocols [30]. The heavy (VH) and light chain (VL) antibody genes were amplified from cDNA generated from the spleens of the five mice immunized with *L. lactis* PilM18. The V_L_ (Vκ and Vλ) and V_H_ PCR reactions were performed separately for each mouse and then pooled. The chimeric heavy (VH-CH1γ) and light (VL-Cκ) cassettes were assembled, and asymmetric SfiI ligation was used to clone into the pComb3× vector as described [31].

The resulting library, which comprised approximately 8 × 10^7^ independently transformed chimeric mouse/human Fab clones, was screened against biotinylated T18.1 immobilized on streptavidin-coupled M-280 Dynabeads (Thermofisher) following published protocols [28]. After four rounds of biopanning with increasing stringency, 190 clones were subject to initial characterization by Fab-phage ELISA using an anti-M13-Horse Radish Peroxidase (HRP) detection antibody (GE Healthcare). Positive clones were subject to DNA sequencing using the Ompseq (5”−AAGACAGCTATCGCGATTGCAG) and Pelseq (5”−ACCTATTGCCTACGGCAGCCG) primers as published [30], which resulted in the identification of 18 Fab clones for initial characterization.

Fab was expressed and purified from the periplasm of non-suppressor *E. coli* Top10F’ cells using Ni^2+^-NTA affinity chromatography. Selected Fab was converted to full IgG by subcloning the mouse variable regions into a human IgG1 backbone and expressed in Epi293F cells (GenScript). All ELISAs were performed following standard protocols [18] in Nunc immunoplates (Thermo Fisher Scientific) coated with the antigen of interest at 5 μg/mL in PBS, pH 7.4 overnight at 4°C. Plates were blocked with PBS supplemented 0.1% (v/v) Tween-20 (PBST) and 5% (w/v) skim milk powder. All wash steps were performed with PBST, and the detected antibodies were as follows: anti-human IgG H+L-HRP (Jackson Immunoresearch, RRID:AB_2340495), anti-mouse IgG-HRP (GE Healthcare, RRID:AB_772210) and anti-rabbit IgG-HRP (Abcam, RRID:AB_955447).

### Flow cytometry

Overnight cultures of *L. lactis* PilM18 were harvested by centrifugation (5000 × g for 5 min) and resuspended in FACS blocking buffer (PBS/3% FBS/5 mM EDTA). The cell suspension was sonicated in a water bath sonicator for 2 min then incubated on ice for 30 min. The blocked cells were harvested by centrifugation and resuspended in FACS buffer (PBS/1% FBS/5 mM EDTA) containing 250 nM chimeric IgG1 and incubated at 4°C or 37°C for 30 min. The cells were washed and then stained with Anti-Human IgG (H+L)-Alexa Fluor 488 (Jackson Immunoresearch) for 30 min on ice. After a final wash, bacteria were analysed by flow cytometry using an LSR II flow cytometer (BD Biosciences) and FlowJo software (FlowJo, LLC).

### Bactericidal assays

Bactericidal assays were performed following published protocols with modifications [32]. M217/T18.1 GAS (M217_11574) was grown in Todd Hewitt broth to an optical density (600 nm) of ~0.2 and diluted to 1:50 for use in the assay and for plating on Todd Hewitt agar plates with 1% yeast extract (THY) to determine CFU. Heparinized whole blood was collected from a healthy adult volunteer (ethics UOA021200), previously screened to confirm sufficient growth of bacteria indicating non-immune status, and used within 2 h of draw. Total assay volume was 300 µl comprising 50 µl bacteria (100–200 CFU), 200 µl of non-immune blood, and 50 µl test sera or antibody. The E3 and H3 monoclonals were included at 5 µg/mL in HBSS media to make up 50 µl equivalent serum volume. Assays were incubated for 3 h at 37°C with 5% CO_2_ and end-over-end rotation before plating on THY agar plates and grown overnight at 37°C with 5% CO_2._ Each assay included pre-immune rabbit sera as a negative control.

### Crystallization, data collection, and structure determination

The E3-T18.1 complex was formed by incubating T18.1 and E3 together at a 2:1 molar ratio for 2 h at room temperature, with the E3-T18.1 complex purified from unbound T18.1 by size exclusion chromatography. Vapour diffusion crystallization trials were conducted using a 288-condition screen comprising the JCSG+/PACT premier screen [33] and the MORPHEUS screen [34]. The E3-T18.1 complex crystals used for X-ray data collection grew by mixing a 1 µl protein solution (88 mg/mL) 1:1 with precipitant (0.3 M diethylene glycol, 0.3 M triethylene glycol, 0.3 M tetraethylene glycol, 0.3 M pentaethylene glycol, 0.1 M MOPS/HEPES-Na pH 7.5, 10% (w/v) PEG 8000 and 20% (v/v) ethylene glycol). Crystals of T18.1 dimer were grown by mixing a protein solution (40 mg/ml) 1:1 with precipitant (0.12 M 1,6-Hexanediol, 0.12 M 1-Butanol, 0.12 M 1,2-Propanediol, 0.12 M 2-Propanol, 0.12 M 1,4-Butanediol, 0.12 M 1,3-Propanediol, 0.1 M Sodium HEPES, 0.1 M MOPS (acid), 10% w/v PEG 8000 and 20% v/v Ethylene glycol). All crystals were grown at 18°C and reached optimum size between 4 and 7 days.

Crystals were flash-cooled in liquid nitrogen without cryoprotectant, and x-ray diffraction data were recorded at the Australian Synchrotron (MX2 beamline). All data sets were integrated using XDS [35] and scaled using AIMLESS [36]. Structures were determined by molecular replacement with Phaser-MR using the T18.1 structure (PDB accession 6N0A) as the model [37] and refined with iterative cycles of manual building in COOT [38] and refinement with REFMAC [39].

The E3-T18.1 complex crystallized in space group *I121* with one monomer of each in the asymmetrical unit. The T18 dimer was also crystallized in space group *I121* with one covalently linked dimer in the asymmetrical unit.

### Data analysis and statistics

Statistical analyses were performed using GraphPad Prism Version 9 (GraphPad Software) and a *P*-value of <0.05 was considered significant. Antibody avidity was estimated as the concentration to reach half-maximum binding (EC_50_) by ELISA and calculated using a five-parameter logistic equation.

## Results

### Selection of T18.1 specific mouse/human chimeric antibodies

**Figure 1.**
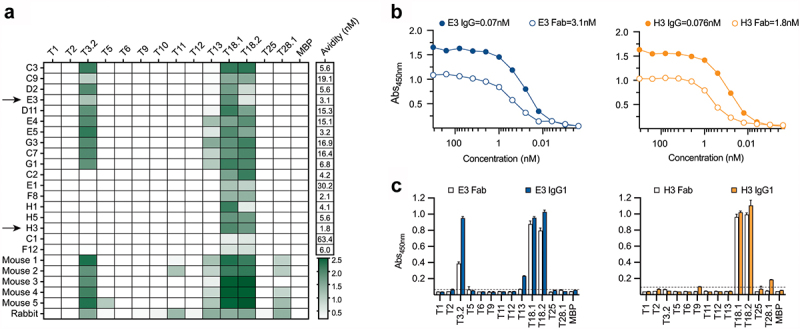
T-antigen specificity of Fab and animal sera. (a) Reactivity of the eighteen Fab and murine and rabbit sera to the T-antigen panel. Sera was diluted at 1: 200 and Fab were diluted to 100 nM, screened by ELISA and absorbances (450 nm) are shown in a colour gradient from white to green. Avidity of the Fab for T18.1 was estimated as the concentration for half-maximum binding (EC50) by ELISA, calculated using an asymmetric sigmoidal, five-parameter logistic equation and shown as an additional column to the right of the heatmap. (b) Comparison of IgG1 and Fab binding to T18.1 by ELISA. The solid lines represent the IgG1 clones while the dotted lines represent the Fab. Estimated avidity of the IgG1 and Fab to T18.1 are indicated. (c) Specificity of IgG1 (solid bars) and Fab (open bars) to the T-antigen panel as measured by ELISA. Dotted line indicates the cut-off (mean+3SD of negative control). Bars represents the mean and standard deviation.

The Fab-phage library, constructed by cloning the heavy (VH) and light chains (VL) from mice vaccinated with *L. lactis* expressing the complete M18/T18 pilus, was screened by phage display against recombinant T18.1 antigen that had been selectively biotinylated at the C-terminus and was immobilized on streptavidin resin. This yielded 18 unique, chimeric mouse/human Fab (Figure 1(a), Supplementary Table S2). Sequence analysis showed all 18 used Vκ light chains and VH CDR3 length ranged between 12 and 15 residues. Binding analysis of the 18 Fab, performed by titration against recombinant T18.1, showed biopanning had identified high avidity Fab with 16/18 having an EC_50_ of <20 nM (Figure 1(a)). Specificity investigation against a panel of recombinant T-antigens highlighted two broad groups; type specific Fab that react with T18.1 and T18.2 only, and cross-reactive Fab that also react with T3.2/T13 (Figure 1(a)). T18/T3 cross-reactivity was a major reactivity pattern observed in our prior analysis of two-domain T-antigens, with T3 typing serum also reacting with T18 antigens [18]. The sera from vaccinated animals, which includes the five mice from which the Fab-phage library was derived, as well as previously described anti-T18.1 rabbit sera [18], illustrate comparable specificity patterns (Figure 1(a)).

Based on the combined sequence, avidity and specificity analysis, two Fab (E3 and H3) were selected for further analysis. The H3 is the highest affinity type-specific Fab, while E3 is the highest affinity cross-reactive Fab. These Fab were converted to full-length chimeric IgG1 by fusion of the mouse variable regions to a human IgG1 backbone. The avidity of the IgG chimeras increased one to two orders of magnitude as expected for bivalent immunoglobulins [40] (Figure 1(b)), and T-antigen specificity was maintained with H3 IgG reacting with T18.1 and T18.2 only, while E3 IgG reacted with T18.1/T18.2 as well as T3.2 (Figure 1(c)).

### Analysis of chimeric IgG interaction with polymerized pilus

**Figure 2.**
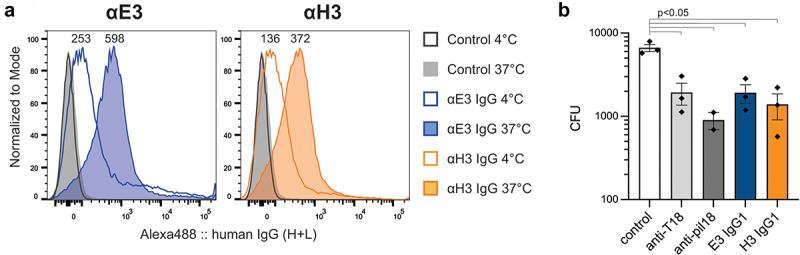
Binding of the IgG1 to T18.1 in the pilus structure. (a). Flow cytometric binding of anti-T18.1 chimeric IgG1 clones and a negative non-binding antibody control (Control) to *L. lactis* PilM18 at 4°C or 37°C shown as a histogram. MFI for each condition is indicated above the peaks. Anti-human IgG Alexa Fluor 488 was used to detect binding and histograms were generated using FlowJo software. Data shown is representative of two independent experiments. (b) Bactericidal activity of vaccinated animal sera (rabbit anti-T18.1 and mouse anti-pilM18) and chimeric IgG1 clones against GAS (M217/T18.1) compared to control naïve rabbit serum. Data combines three independent experiments and is shown as mean ± SEM. For statistical analysis CFU data were log transformed and analysed by one-way ANOVA with Holm-šídák’s multiple comparisons test.

Flow cytometry and bactericidal assays were utilized to confirm that the IgG chimeras were able to recognize polymerized T18.1 within the pilus structure expressed on the surface of the surrogate food-grade bacterium *Lactococcus lactis*. For flow cytometry, binding to *L. lactis* PilM18 was initially analysed at 4℃ as per standard protocols. At this temperature, only weak binding was observed for E3 and H3 as indicated by a moderate mean fluorescence intensity (MFI) shift compared to control (Figure 2(a)). However, when IgG chimera binding was analysed after incubation of the *L. lactis* PilM18 at 37°C, E3 and H3 showed a >2-fold increase in MFI (Figure 2(a)). This marked increase in binding indicated that the E3 and H3 epitopes were more accessible at physiological body temperatures than at 4°C.

Bactericidal assays were performed using the IgG1 chimeras against the *emm*217 GAS strain that carries *tee*18.1, and thus expresses the T18.1 antigen within the pilus structure. As positive controls, sera from the mice vaccinated with *L. lactis* PilM18 and the rabbit vaccinated with recombinant T18.1 were able to induce killing in the indirect bactericidal assay (Figure 2(b)). Similarly, both E3 and H3 IgG induced killing compared to the negative control (pre-immune rabbit sera). These assays were performed at 37°C, and given that opsonophagocytic killing relies on phagocytic cells recognizing antibodies bound to GAS (opsonization) for uptake, this is further evidence that the IgG chimeras recognize T18.1 within the pilus structure.

### Epitope mapping of T-antigen antibody binding

**Figure 3.**
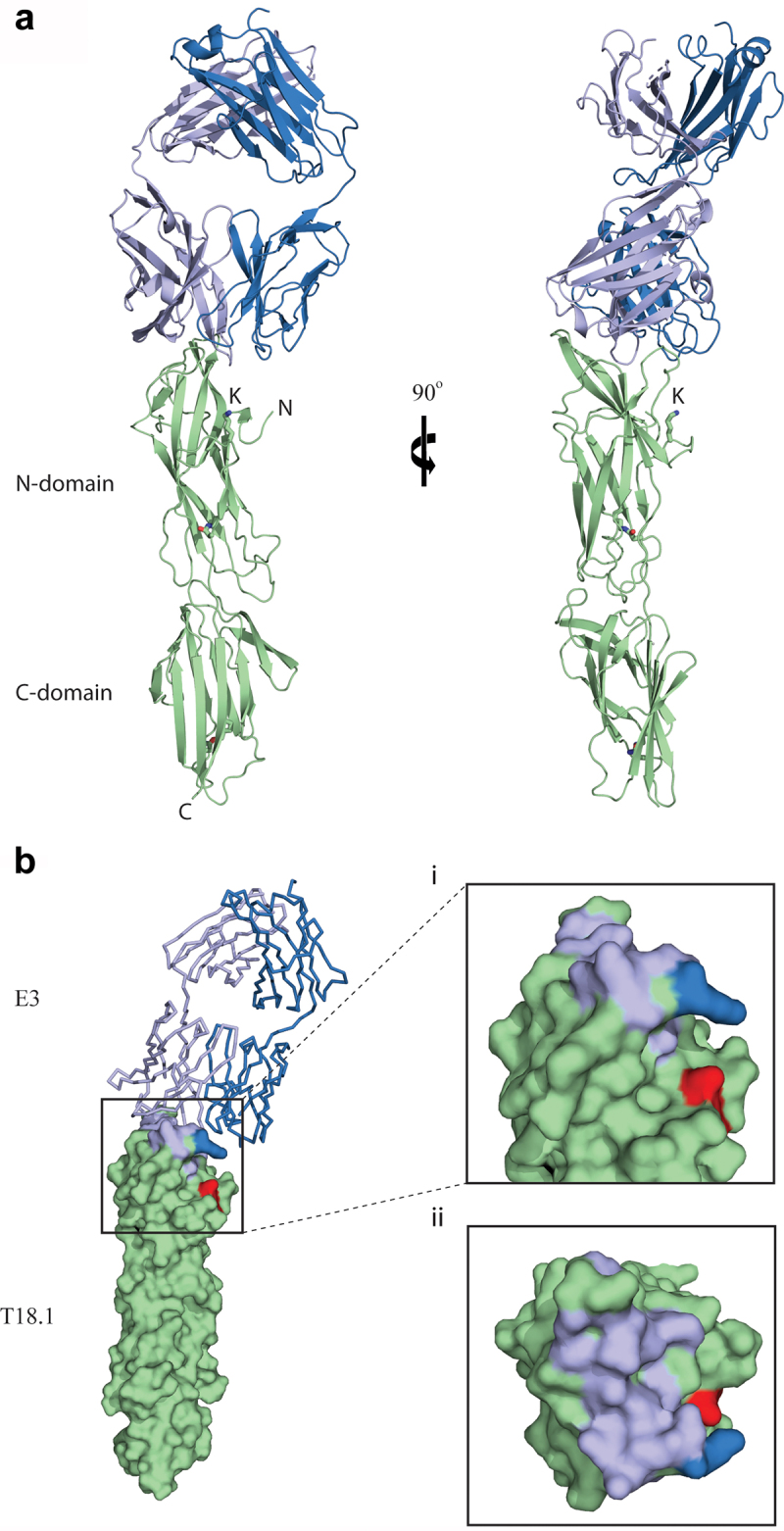
The structure of the E3-T18.1 complex. (a) The E3 Fab heavy chain (light blue) forms the majority of the binding interface with only one loop of the light chain (dark blue) making contact with the T18.1 N-terminal domain (green). The pilin lysine (residue Lys 146) that would be covalently linked to the sortase motif at the C-terminus of the next pilin is shown. (b) The E3 epitope mapped onto the surface of T18.1. (i) The T18.1 residues that make up the E3 epitope are shown in light blue (E3 heavy chain contacts) and dark blue (E3 light chain contacts). The pilin lysine is shown in red. (ii) A “top down” view of the epitope highlights that E3 binds across the top of the pilin N-domain and almost entirely via the Fab heavy chain. (*N* = N-terminus of T.18.1, C = C-terminus, K = pilin lysine).

The epitope for E3 was determined by x-ray crystallography, with the E3-T18.1 complex structure solved by molecular replacement and refined to a resolution of 1.90 Å (*R* 21.9%; *R*free 25.0: PDB code 8F5N) (Supplementary Table S3). As previously observed [18], T18.1 has a two-domain structure, and there is excellent electron density for the intramolecular isopeptide bonds within each Ig-like domain. The light chain of E3 is also fully modelled, and while the heavy chain has two short loop regions in CH1γ with uninterpretable electron density (residues 134–141 and 195–198), these are distant from the T18.1 interaction site. The E3 Fab binds to the top of the T18.1 N-domain (Figure 3a), with the epitope comprised 14 residues that interact with 16 E3 residues in the paratope (Supplementary Figure S1). The heavy chain is the main contributor to binding interactions including S31, F32, and Y33 in CDR1; N52, N55, A58, and N59 in CDR2; and F100, Y101, Y102, G103, and W105 in CDR3. In contrast, only four paratope residues are located in the light chain and all of these are found in CDR3 (V91-L94).

The E3 epitope residues map across the top of the N-domain of T18.1 (Figure 3(b)), and the interaction includes nine hydrogen bonds and a network of 80 non-bonding interactions (Suplementary Figure S1). The N125 in T18.1 is likely a key determinant of binding as it forms a network hydrogen bond with Y101 in CDR3 and S31 in CDR1 of the E3 heavy chain with additional hydrogen bonding to CDR1 and CDR3 through a well-defined water molecule. The E3-T18.1 epitope was somewhat unexpected as the top of the T18.1 N-domain directly overlaps with the predicted inter-pilin interface. That is, the base of the C-domain of the next T-antigen slots into a shallow V-shaped groove at the top of the N-domain in the polymerized pilus (Young et al., 2019, Kang et al., 2007). When T-antigen monomers are incorporated into pilus structures this would bury the N-C-domain interface, suggesting that the E3 Fab would compete with the C-domain of the proceeding pilin monomer when binding to the polymerized pili. The increased binding of E3 to the polymerized pilus at 37°C (Figure 2(b)) suggests that these epitopes are exposed in pili at physiological temperatures and that there must be enough flexibility between monomers to not only expose the epitope but also accommodate the bulk of E3 once bound.

**Figure 4.**
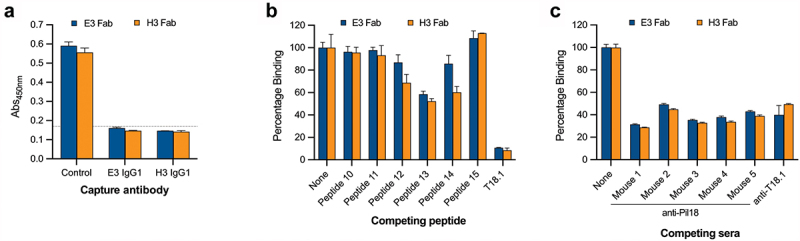
Epitope characterization of the Fab. (a) Capture ELISA in which recombinant T18.1 was captured by chimeric IgG1 coated wells. Fab binding to captured T18.1 was detected using anti-HA-HRP. Binding is compared with a positive (no capture) T18.1 control, and the dotted line represents the negative control cut-off (no fab, mean+3SD). (b) Competition ELISA for the Fab and the epitope spanning peptides. Fab were pre-incubated with each peptide and percentage binding to T18.1 was calculated by comparison with a non-peptide control. (c) Competition ELISA for the Fab with sera from the vaccinated animals. Fab were added together with each antisera and percentage binding was calculated by comparison with a control (1% BSA). Bars represent mean plus SD.

Capture ELISA performed with E3 and H3 IgG chimeras and Fab showed that capturing T18.1 with each antibody inhibited binding of the other (Figure 4(a)), suggesting that the epitope for the two antibodies overlaps. Further confirmation was provided by ELISA with overlapping 15-mer peptides derived from the broader epitope region (aa91–155) where peptides 12, 13, and 14, which contain seven of the 14 epitope residues identified in the cocrystal structure, inhibit binding of both E3 and H3 Fab to T18.1 (Figure 4(b), Supplementary Figure S2). There were some differences in the ability of each peptide to inhibit Fab-T18.1 binding, with peptide 14 blocking more for H3 Fab than for E3, suggesting that while the epitopes overlap, they are not identical. This is consistent with the differing T-antigen reactivity profiles of the two antibodies with E3 cross-reacting with T3.2, while H3 is type specific (Figure 1(a,c)) and the sequence differences along the broader epitope region between T18.1/T18.2 and T3.2 (Supplementary Figure S2).

To confirm that animals vaccinated with *L. lactis* PilM18 produce antibodies that bind to the same region as the Fab identified by phage display, competition ELISA was performed. The PilM18 sera markedly reduced binding of E3 and H3 Fab to T18.1, as did serum from a rabbit vaccinated with monomeric recombinant T18.1 protein (Figure 4(c)). This indicates that animals vaccinated with both monomeric and polymerized T-antigens produce antibodies that bind to the top of the N-domain, suggesting this is a highly immunogenic region of the pilus.

### Structural analysis of the T-antigen interface within polymerized pilus

The solvent buried area of the E3-T18.1 interface, as calculated by PDBePISA, is ~840 Å^2^. This is twice that for the predicted interface between T1 monomers (~381 Å^2^ deduced from T1 crystalline packing [21]). To more accurately define the interface between T-antigens within the pilus, a covalently linked T18.1 dimer was generated in which the C-terminal sortase motif of one monomer was fused to the N-terminus of the following monomer with a flexible (glycine)_3_ linker. This produced a dimer covalently linked in a manner similar to the sortase catalyzed wild-type (WT) found in the polymerized pili, given the close proximity of the N-terminus to the pilin lysine (K) in the T18.1 structure (Figure 3(a)) and as published [18]. The T18.1 dimer was crystallized and the structure refined using data to a resolution of 2.29 Å (*R* 19.3%; *R*free 25.4: PDB code 8F70) (Supplementary Table S3), with interpretable electron density for the entire dimer except for the flexible glycine linker between monomers. The sortase motif of the first monomer is positioned in the peptide-binding groove in the N-terminal domain of the second monomer as anticipated, though electron density is poor at the C-terminal end of the motif (residues 284–288). In the structure, there are no contacts made between the sortase motif and the binding groove of the second monomer, but in the WT pilus it is possible that Pro 286 may be more closely positioned and form non-polar contacts with Tyr 406 or Tyr 419 (Tyr 122 and Tyr 135 in WT).

**Figure 5.**
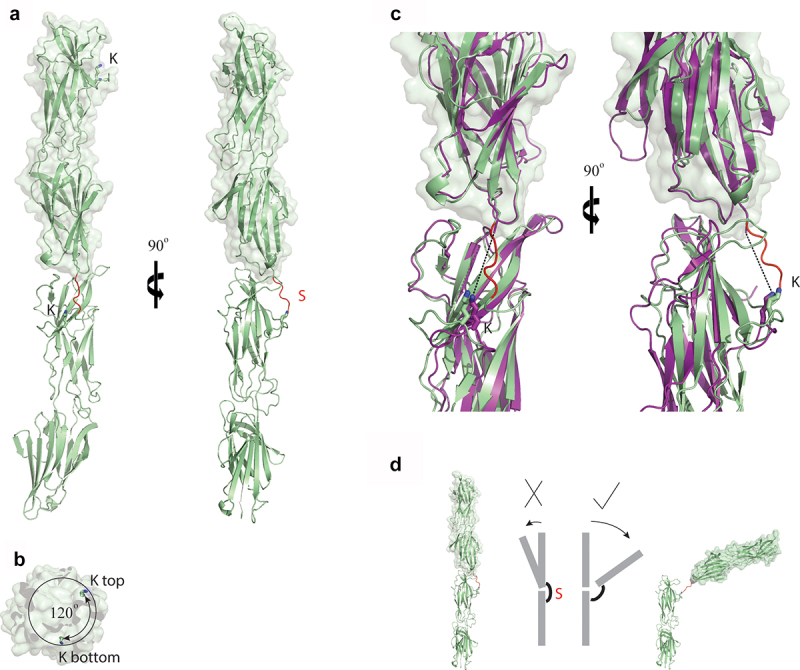
Structural analysis of the T-antigen interface within the polymerized pilus. (a) Ribbon diagram of the T18.1 dimer show that pilins stack end-on-end. The electron density of the sortase motif (S) connecting the two pilins is only partially interpretable. (b) Top view of the T18.1 dimer showing a 120° rotation between each pilin along the long axis, which is identical to that measured in T1 antigen crystal packing [21]. (c) The interface between the two T18.1 pilin monomers (green) is virtually identical to the T1 crystal packing interface (magenta), as is the distance between the pilin lysine (k) and the C-terminus of the next pilin (dashed line). (d) The small interface supports a model in which T-antigen inter-pilin contacts are relatively weak, potentially functioning like a knee joint whereby the pilin flexes in only one direction as the covalent isopeptide bond prevents bending in the opposite direction, or complete disassociation. The 120° rotation between each successive pilin allows 360° flexibility along the length of the pilus. (T18.1 = green, T1 = magenta, K = pilin lysine, S = sortase motif).

**Figure 6.**
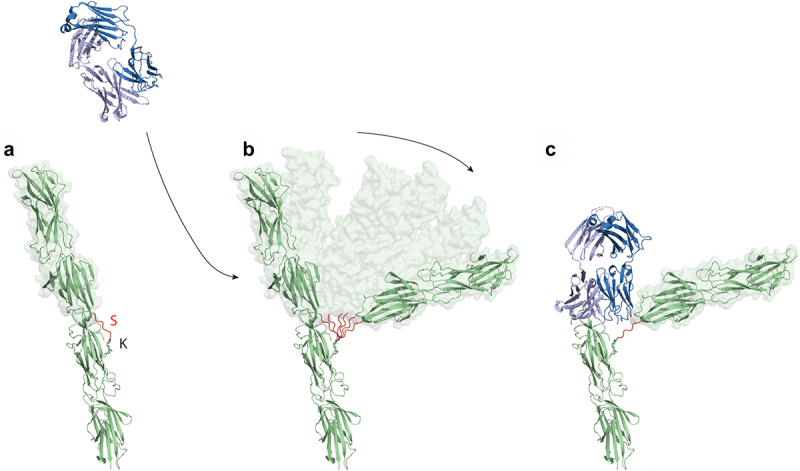
The binding of the E3 Fab to the polymerized T18.1 pilus indicates significant flexibility between T-antigen monomers. (a) Ribbon diagram showing T18.1 dimer structure depicting the polymerization of T18.1 subunits in the pilus. The inter-domain sortase linker (S) is highlighted in Red. The final threonine in this linker (TQVPT) is covalently linked to the pilin lysine (K) in the N-domain of the proceeding pilin via a Sortase C catalysed isopeptide bond. (b) The small interface between pilins suggest they may temporarily disassociate, bend away from the vertical axis, which allows E3 to bind. (c) The top T18.1 subunit would be displaced by the E3 Fab, but is still covalently linked to the next pilin in the pilus.

The T18.1 dimer stacks end-on-end with a 120° rotation along the long axis (Figure 5(a,b)), which is identical to the pilin rotation in the T1 crystal packing [21]. The interface between the two T18.1 monomers is virtually identical to the T1 interface (Figure 5(c)), with a buried surface area of 488 Å [2]. This relatively small interface implies that T-antigen contacts are relatively weak and potentially transient, providing a point for motion within the polymerized pilus. Examination of the structure suggests that the pilin interface could function like a knee joint whereby the pilin can bend away from the vertical axis in the direction of the sortase catalyzed covalent inter-pilin isopeptide bond (Figure 5(d)). The covalent, sortase catalyzed, inter-pilin isopeptide bond would prevent complete disassociation, and any flexing in the opposite direction, enabling the potential mechanism by which E3 binds to the N-domain of a T18.1 monomer within the pilus (Figure 6).

## Discussion

This study has combined vaccination with a polymerized pilus and Fab-phage display technology to identify T-antigen specific monoclonal antibody fragments that target an immunodominant region on the N-terminal region of T18.1. This representative two-domain T-antigen (FCT-3/4 region) is found in *emm*18, *emm*49, and *emm*217 strains, which have been associated with serious invasive infections and acute rheumatic fever globally [41–45]. The immunodominance of the N-terminal region within the N-terminal domain may have implications for vaccine design, in particular, the TeeVax candidate that has the N- and C-terminal domains of selected T-antigens alternating within the multivalent recombinant vaccine [27]. While the N-terminal domain of T18.1 has been incorporated in TeeVax, further investigation is required to determine if there is domain-specific immunodominance within other T-antigens included in this preclinical vaccine.

Previous structural and immunological analyses of T-antigens have shown that, despite heterogeneity in surface-exposed residues between T-antigens, cross-reactive epitopes exist between selected T-types [18,25,29]. Identification of the E3 epitope in this study provides molecular insight into the basis of cross-reactivity between T18.1/T18.2 and T3 and T13 antigens, with key epitope residues conserved between these T-types. In particular, the N125 residue in T18.1, which forms a network of hydrogen bonds with the E3 heavy chain, is also present in T3 and T13. However, the identification of H3 Fab in this study, which reacts with T18.1/T18.2 and not T3 or T13, also illustrates the presence of T-type specific epitopes. That the H3 epitope overlaps with that of E3 suggests there are multiple, overlapping epitopes in the N-terminal domain that differ in the level of sequence similarity between related T-antigens. This highlights the complexity of predicting T-type specific antibody responses based on sequence alone, in keeping with the cross-reactivity of selected T-typing antisera [25] and a reflection of these antigens having a high level of surface sequence divergence distributed along their length that shields a highly conserved structure [18].

It is the lengthwise distribution of sequence variation, as well as the mechanism of polymerization whereby one pilin subunit is covalently linked to the next end-on-end [13], which made the identification of a cross-reactive, protective epitope at the N-terminal region surprising. The interface between two pilin subunits, deduced from the dimeric T18.1 structure in this study, as well as from the crystal packing of the T1 structure [21], clearly show that the N-terminal epitope region of the E3 and H3 Fab would be buried by the C-terminal domain of the next pilin subunit, explaining the relatively modest Fab-pilus binding observed at 4°C. However, the marked increase in Fab binding at 37°C suggests that these epitopes are more accessible at physiological temperatures, and that there must be movement between pilin subunits to enable Fab binding. The covalent, inter-pilin isopeptide bond would restrict flexing in one direction, suggesting a knee-joint like motion with enough flexibility between monomers to not only expose the epitope but also accommodate a Fab molecule. As each pilin monomer is rotated 120 degrees to the previous one, the pilus fibre as a whole would have the ability to flex in any direction (analogous to a rope) rather than being a rigid structure. Indeed, electron microscopy has also shown that the pilus fibres exhibit some “give” with immunogold labelled pilus shafts displaying some curvature [12,46]. During infection, this motion or ability to bend likely benefits the process of adhesion, where the GAS pilus plays a pivotal role by binding to extracellular matrix components such as fibronectin and collagen [14]. Having motion and flexibility in the pilus backbone could be advantageous to enable positioning of the tip adhesion (AP1) to these extracellular matrix components at preferred angles for binding.

While there has been a lack of studies investigating temperature-dependent, structural changes in epitope accessibility in large bacterial assemblies such as pili, this has been elegantly demonstrated using cryo-electron microscopy techniques for viral envelope proteins in the context of the virion [47–49]. In particular, the dengue virion has been shown to undergo major rearrangement and “breathing” to expose specific epitopes on the envelope protein at 37°C not available at lower temperatures [48], and epitope accessibility has been identified as a significant factor in determining the protective capacity of antiviral antibodies [50, 51]. The major impact of temperature on antibody-pilus binding observed in this study highlights the importance of considering physiological conditions when investigating functional antibody responses for bacterial pathogens, particularly in the context of opsonization and phagocytosis. Though only a single T-type has been investigated, the strong structural homology between two-domain T-antigens [18] implies that this temperature dependent, mechanistic flexing is conserved throughout GAS pili. Further studies focused on understanding the exposure of protective epitopes this flexing allows will be important in determining pilin-based, vaccine-induced protection at the molecular level.

## Supplementary Material

Supplemental Material

## Data Availability

Atom coordinates and structure factors have been deposited in the Worldwide Protein Data Bank (wwPDB) with PDB accession numbers 8F5N for E3-T18.1 and 8F70 for T18.1 dimer. The authors confirm that the remaining data supporting the findings of this study are available within the article. Additional data are available from the corresponding author [NJM] upon reasonable request.
