# Supplementary material for: Identification of an immunodominant region on a group A Streptococcus T-antigen reveals temperature-dependent motion in pili
Source: Virulence. 2023 Feb 21;14(1):2180228. doi: 10.1080/21505594.2023.2180228 (PMC9980535; doi:10.1080/21505594.2023.2180228)

**Supplementary Information**

**Supplementary Table 1.** Properties of the T18.1 overlapping peptide library.

| Peptide | Sequence* | Length | Position | Molecular Weight |
| --- | --- | --- | --- | --- |
| 1 | ETAGVIDGSTLVVKK | 15 | 1->15 | 1516.74 |
| 2 | LVVKKTFPSYTDDKV | 15 | 11->25 | 1740.02 |
| 3 | TDDKVLMPKADYTFK | 15 | 21->35 | 1772.05 |
| 4 | DYTFKVEADDNAKG**K** | 15 | 31->45 | 1700.81 |
| 5 | NAKG**K**T**KDGLD**IKPG | 15 | 41->55 | 1541.74 |
| 6 | **D**IKPGVIDGLENTKT | 15 | 51->65 | 1599.79 |
| 7 | ENTKTIHYGNSDKTT | 15 | 61->75 | 1708.80 |
| 8 | SDKTTAKEKSVNFDF | 15 | 71->85 | 1716.86 |
| 9 | VNFDFANVKFPG**V**GV | 15 | 81->95 | 1609.82 |
| 10 | PG**V**GVYRYTVSEVNG | 15 | 91->105 | 1596.75 |
| 11 | SEVNGNKAGIAYDSQ | 15 | 101->115 | 1552.60 |
| 12 | AYDSQQWTVDV**Y**V**VN** | 15 | 111->125 | 1786.90 |
| 13 | V**Y**V**VNR**ED**G**G**F**EA**K**Y | 15 | 121->135 | 1745.90 |
| 14 | **F**EA**K**YIVSTEGGQSD | 15 | 131->145 | 1630.73 |
| 15 | GGQSDKKPVLFKNFF | 15 | 141->155 | 1711.96 |
| 16 | FKNFFDTTSLKVTKK | 15 | 151->165 | 1804.11 |
| 17 | KVTKKVTGNTGEHQR | 15 | 161->175 | 1682.88 |
| 18 | GEHQRSFSFTLLLTP | 15 | 171->185 | 1732.97 |
| 19 | LLLTPNECFEKGQVV | 15 | 181->195 | 1689.98 |
| 20 | KGQVVNILQGGETKK | 15 | 191->205 | 1598.83 |
| 21 | GETKKVVIGEEYSFT | 15 | 201->215 | 1686.88 |
| 22 | EYSFTLKDKESVTLS | 15 | 211->225 | 1746.94 |
| 23 | SVTLSQLPVGIEYKV | 15 | 221->235 | 1632.91 |
| 24 | IEYKVTEEDVTKDGY | 15 | 231->245 | 1788.93 |
| 25 | TKDGYKTSATLKDGD | 15 | 241->255 | 1599.71 |
| 26 | LKDGDVTDGYNLGDS | 15 | 251->265 | 1568.60 |
| 27 | NLGDSKTTDKSTDEI | 15 | 261->275 | 1623.69 |
| 28 | STDEIVVTNKRDTQV | 15 | 271->285 | 1704.85 |
| 29 | RDTQVPT | 7 | 281->287 | 815.88 |

*Residues that make up the αE3 epitope are in bold.

**Supplementary Table 2.** Sequence analysis of the 18 Fab clones identified by phage display.

| Fab clone | Nucleic acid identity† | | CDR Length‡ | | CDR sequences | |
| --- | --- | --- | --- | --- | --- | --- |
|  | VL (% / gene*allele) | VH (% / gene*allele) | VL | VH | VL CDR3 | VH CDR3 |
| C2 | 99.25 / IGKV4-59*01 | 94.79 / IGHV1-26*01 | [5.3.9] | [8.8.14] | QQSKEVPFT | ARRFTNYG.RSYFDY |
| C3 | 97.13 / IGKV3-2*01 | 96.53 / IGHV1S5*01 | [10.3.9] | [8.8.13] | QQSKEVPFT | ARGY.YDG.SHYFDY |
| C9 | 83.15 / IGKV3-2*01 | 95.49 / IGHV1S5*01 | [6.3.9] | [8.8.13] | QQSKEVPFT | TRGF.YDG.SHYFDY |
| D2 | 96.28 / IGKV17-121*01 | 94.44 / IGHV1S5*01 | [6.3.9] | [8.8.13] | LQSDNLPFT | TRGY.YDG.SHYFDY |
| E1 | 99.62 / IGKV6-23*01 | 95.14 / IGHV5-12-1*01 | [6.3.9] | [8.8.15] | QQYSSYPYT | ARHKERHDGDYHFDV |
| **E3** | 96.76 / IGKV10-96*01 | 93.75 / IGHV1S81*02 | [6.3.9] | [8.8.13] | QQTNTLPFT | TRGF.YYG.HWYFDV |
| E4 | 95.17 / IGKV10-96*01 | 91.67 / IGHV1S5*01 | [6.3.9] | [8.8.13] | QQVETLPFT | ARGY.YDG.SHYFDY |
| E5 | 97.58 / IGKV3-2*01 | 94.79 / IGHV1S5*01 | [10.3.9] | [8.8.14] | QQSKEVPFT | ARSY.YSKYGHYFDY |
| F8 | 96.19 / IGKV3-12*01 | 96.53 / IGHV1S5*01 | [10.3.9] | [8.8.13] | QDSGELPLT | TRGF.YDG.SHYFDY |
| G3 | 97.44 / IGKV10-96*01 | 95.83 / IGHV1S55*01 | [6.3.9] | [8.8.13] | QQVNTLPFT | ARGF.YDG.YFYFDY |
| H1 | 99.64 / IGKV6-17*01 | 96.18 / IGHV1S5*01 | [6.3.9] | [8.8.15] | QQHYSTPYT | VRGYYSDYEIWKFDY |
| **H3** | 98.88 / IGKV4-72*01 | 94.79 / IGHV1-9*01 | [5.3.9] | [8.8.15] | QQWSSNPPT | GRRRVYYYEGSYVDY |
| H5 | 99.62 / IGKV4-72*01 | 94.44 / IGHV1S5*01 | [5.3.9] | [8.8.15] | QQWSSNPPT | ARRRVYYYDGSYVDY |
| G1 | 94.07 / IGKV6-13*01 | 93.75 / IGHV1S130*01 | [6.3.9] | [8.8.13] | QNDYSYPFT | ARGF.YGG.FFYFDY |
| C1 | 97.12 / IGKV6-15*01 | 97.19 / IGHV2-2*01 | [6.3.9] | [8.7.12] | QQYNSYPYT | ARN..WGLLGR.MDY |
| C7 | 97.04 / IGKV10-96*01 | 96.53 / IGHV1S5*01 | [6.3.9] | [8.8.13] | QQVNTLPLT | ARGF.YDG.SHYFDY |
| D11 | 98.56 / IGKV10-96*01 | 92.71 / IGHV1S5*01 | [6.3.9] | [8.8.13] | QQTNTLPFT | ARGF.YDG.SHYFDY |
| F12 | 94.81 / IGKV10-96*01 | 97.57 / IGHV1S5*01 | [6.3.9] | [8.8.13] | QQVETLPFT | ARGY.YDG.SHYFDY |

†Percentage identity to the germline gene as calculated by IMTG/V-QUEST.

‡CDR length of [CDR1.CDR2.CDR3] as determined by IMTG/V-QUEST.

**Supplementary Table 3.** Collection and analysis parameters for E3-T18.1 and T18.1 dimer

| Collection Statistics | αE3-T18.1 | T18.1 dimer |  |
| --- | --- | --- | --- |
| PDB code  Wavelength (Å) | 8F5N  0.95374 | 8F70  0.95374 |  |
| No. of images | 3600 | 2735 |  |
| Oscillation angle (°) | 0.1 | 0.1 |  |
| *Resolution range (Å) | 41.96 -1.90 (1.94 – 1.90) | 47.58 – 2.29 (2.37 – 2.29) |  |
| *Total no. of observations | 451654 (29469) | 145410 (13215) |  |
| *Unique reflections | 67091 (4288) | 27529 (2540) |  |
| *Redundancy | 6.7 (6.9) | 5.3 (5.2) |  |
| Space group | I 1 2 1 | I 1 2 1 |  |
| Unit-cell axial lengths (Å)  angles (°) | a=126.57 b= 41.51 c= 172.35  *α*= 90.00, *β* = 110.10, *γ* = 90.00 | a= 34.29 b= 46.98 c= 380.63  *α*= 90.00, *β* = 90.12, *γ* = 90.00 |  |
| *Completeness (%) | 100 (100) | 99.4 (95.0) |  |
| *Mean I/σ(I) | 8.6 (0.8) | 7.2 (1.7) |  |
| *R_merge_ (%) ^†^ | 0.095 (2.243) | 0.144 (0.987) |  |
| *CC(1/2) ^ϕ^ | 0.998 (0.432) | 0.993 (0.714) |  |
| Refinement statistics |  |  |  |
| *Resolution range (Å) | 41.96 -1.90 (1.94 – 1.90) | 47.58 – 2.29 (2.37 – 2.29) |  |
| Molecules per A.U. | 1 | 1 |  |
| Solvent content (%) | 52 | 50.2 |  |
| R_work_ /R_free_ (%) ^‡^ | 21.9/25.0 | 19.3/25.4 |  |
| Protein atoms | 5289 | 4303 |  |
| Water molecules | 302 | 213 |  |
| RMS deviation from ideal bond length(Å) angle (°) | 0.007 0.90 | 0.007  1.49 |  |
| Average B-factor (Å^2^) |  |  |  |
| Protein | 53.1 | 40.2 |  |
| Water | 51.7 | 37.9 |  |
| Ramachandran Most Favoured (%) | 97.1 | 96.0 |  |
| Ramachandran Outliers (%) | 0.15 | 0.0 |  |
| *Numbers in parentheses for outermost shell. ϕ Mn(I) half-set correlation CC(1/2) as calculated by Scala.  †$R_{merge}=\sum_{hkl} {\sum_{i} \left\vert I_{i}\left( hkl \right)-\left\langle I\left( hkl \right) \right\rangle\right\vert}/{\sum_{hkl} \sum_{i} I_{i}\left( hkl \right)}$. ‡ $R_{work}$ and $R_{free}=\sum\left\vert\left\vert F_{obs} \right\vert-\left\vert F_{calc} \right\vert\right\vert/{\sum\left\vert F_{obs} \right\vert}$, where *R_free_* was calculated over 5 % of amplitudes that were chosen at random and not used in refinement. | |  |  |

**Supplementary Figure 1.** ProFunc calculation of the E3 epitope on T18.1. Hydrogen bonds are shown in blue. Non-bonding interactions are shown in orange where the width of the striped line is proportional to the number of atomic contacts. Residue colours: Blue = positive; Red = negative; Green = neutral; Grey = aliphatic; Purple = aromatic.


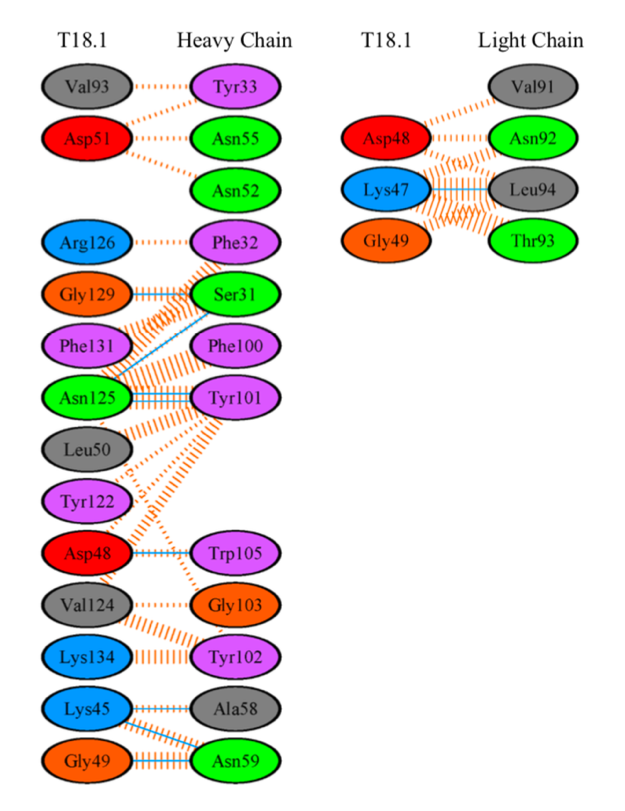


**Supplementary Figure 2.** Protein alignment of the 35-residue immunogenic region (A111-D145) in all 2 domain T antigens in the panel. The residues are numbered according to the sequence of T18.1. The T antigens that the E3 antibody cross-reacts with are bracketed. Residues which are part of the E3 epitope on T18.1 are shown in red boxes. The coloured lines represent peptide 12 (blue), peptide 13 (teal) and peptide 14 (green) from the overlapping peptide library. The alignment was generated using ClustalW and ESPript.


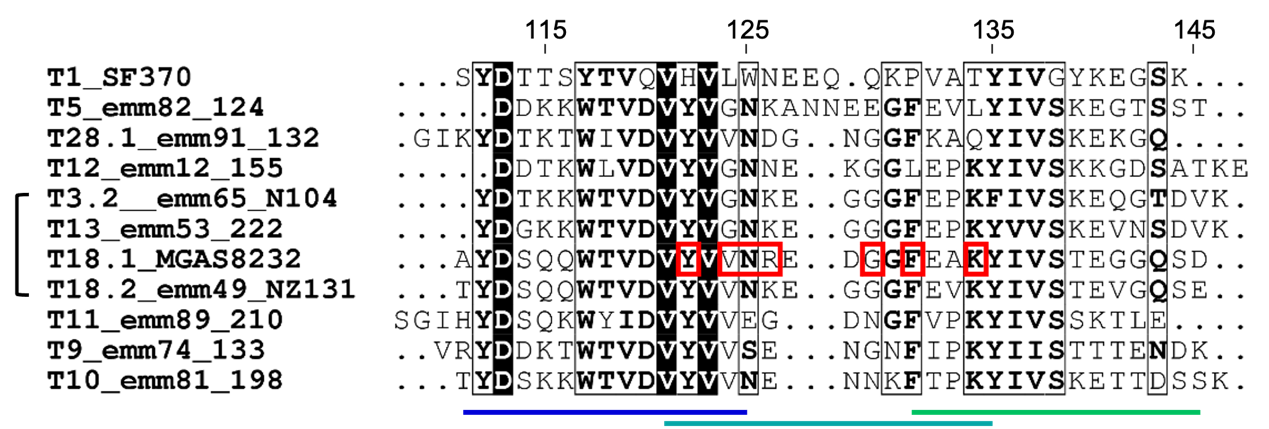

Supplement: Supplemental Material [file KVIR_A_2180228_SM6068.docx]
